# Human Actions Analysis: Templates Generation, Matching and Visualization Applied to Motion Capture of Highly-Skilled Karate Athletes

**DOI:** 10.3390/s17112590

**Published:** 2017-11-10

**Authors:** Tomasz Hachaj, Marcin Piekarczyk, Marek R. Ogiela

**Affiliations:** 1 Institute of Computer Science, Pedagogical University of Krakow, 2 Podchorazych Ave, 30-084 Krakow, Poland; marcin.piekarczyk@up.krakow.pl; 2 AGH University of Science and Technology, Cryptography and Cognitive Informatics Research Group, 30 Mickiewicza Ave, 30-059 Krakow, Poland; mogiela@agh.edu.pl

**Keywords:** motion capture, signal averaging, template generation, kinematic, quaternions, karate, signal processing, dynamic time warping, barycenter averaging

## Abstract

The aim of this paper is to propose and evaluate the novel method of template generation, matching, comparing and visualization applied to motion capture (kinematic) analysis. To evaluate our approach, we have used motion capture recordings (MoCap) of two highly-skilled black belt karate athletes consisting of 560 recordings of various karate techniques acquired with wearable sensors. We have evaluated the quality of generated templates; we have validated the matching algorithm that calculates similarities and differences between various MoCap data; and we have examined visualizations of important differences and similarities between MoCap data. We have concluded that our algorithms works the best when we are dealing with relatively short (2–4 s) actions that might be averaged and aligned with the dynamic time warping framework. In practice, the methodology is designed to optimize the performance of some full body techniques performed in various sport disciplines, for example combat sports and martial arts. We can also use this approach to generate templates or to compare the correct performance of techniques between various top sportsmen in order to generate a knowledge base of reference MoCap videos. The motion template generated by our method can be used for action recognition purposes. We have used the DTW classifier with angle-based features to classify various karate kicks. We have performed leave-one-out action recognition for the Shorin-ryu and Oyama karate master separately. In this case, 100% actions were correctly classified. In another experiment, we used templates generated from Oyama master recordings to classify Shorin-ryu master recordings and vice versa. In this experiment, the overall recognition rate was 94.2%, which is a very good result for this type of complex action.

## 1. Introduction

Human movements and actions analysis is a very interesting and up to date subject of wide research. This is mostly within the scope of interest of biomechanics, sport, medicine and rehabilitation. Human movements can be analyzed by various methods, for example by kinetic modeling [1,2,3], kinematic [3,4,5,6,7,8], electromyography [4,6,9,10,11], gait and posture analysis [12] and pattern recognition [13]. Quite often, those evaluations require statistical analysis of data from several measurements of the same action. Due to this, signal averaging and smoothing are common approaches. Those signal processing techniques are very often used in motion capture (MoCap) for missing sample complementing (estimating missing marker positions), averaging data from many captures of the same activity or removing the high frequency noises (smoothing) of the overall solution [14,15,16,17,18,19]. Averaging of several performances of one action allows us to filter out small deviations that are not important in the whole movement. It can also be applied for the generation of action templates that can be compared with other signals of the same activity, in order to detect similarities and differences between them. Often, those types of analyses, for example in sport sciences, are modeled as one-dimensional movement [4,6,20,21,22,23] or two-dimensional path analysis problems [24].

There are already papers dealing with the application of DTW in motion capture data analysis and template generation. In [25], the authors generate motions templates (MT), which are a set of relational motion features that describe geometric relations between specified points of a pose. Those MT are than used in the classification process with DTW. After changing the DTW cost function, it might be adapted to operate on higher dimensional signals, for example quaternions [26]. The initial research on using quaternions and DTW for the full-body classification problem (without a description of how to create dedicated MT) is presented in [27]. DTW with quaternion-based distance and the nearest neighbor classification is used in the gait classification problem in [28,29]. In [30], an approach based on DTW and Hermite curve interpolation is used to normalize the length of motion sequences. In [31], the authors propose an algorithm that allows matching two discrete time functions, of which one is the original and the other can be considered a not so accurate copy with modified time, noise and a rotated coordinate system using quaternions, principal components analysis (PCA) and DTW. In [32], the authors use gyroscope sensors and various pattern classification techniques (among them DTW) to assign MoCap to one of the eight classes. In [33], the authors perform analysis of golf swings using a single motion sensor that is comprised of gyroscopes and accelerometers. Applying the PCA to the reference observations of properly performed swings, the PCA components of acceptable swing motion deviations are established. There are also many papers dealing with human action recognition using various acquisition methods and classification approaches, for example [34,35,36].

**Figure 1:**
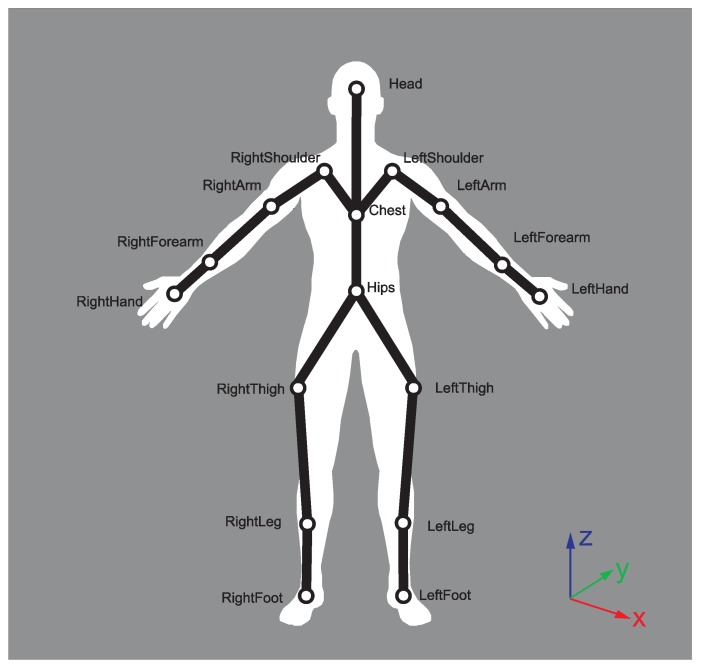
The hierarchical kinematic model that we used in our modeling. The root node of the model is the hips joint. The local coordinate system of each joint is right-handed. In the bottom left part of the image is the global right-handed coordinate system.

The direct inspiration for this research was the fact that to our best knowledge, there were no published papers that deal with the problem of the three-dimensional whole body MoCap data averaging that comes from multiple recordings of the same activity. Due to this fact, we did not find an appropriate approach to which to compare the proposed method. However, we have evaluated our approach in the role of template generation and matching for inertial sensor-based action recognition. What is more, our averaged data can be visualized using a kinematic model as the one presented in Figure 1 and presented directly to the expert. The aim of this paper is to introduce and evaluate the novel method of template generation, matching, comparing and visualization applied to motion capture (kinematic) analysis of motion capture data, which is a valuable extension of already published works [37]. The algorithm in [37] is not aware of quaternion dual mapping, and due to this fact, some three-dimensional rotations represented with quaternions could not be wrapped correctly. The algorithm proposed in this paper fixes this issue. Furthermore, this paper introduces mathematical fundaments of the averaging and smoothing algorithm, while the previous one proposes only one possible implementation. We also present the methodology of DTW-matched results visualization in 3D (DTW signal mappings; see Algorithm 3, Figure 3), which is especially useful when one can manually rotate and observe this mapping from various angles. Furthermore, we introduce a very useful DTW signal distance plot (see Section 2.5 and Figure 4) that allows identifying the corresponding parts of actions that are the least and the most similar to each other. Those two methods of DTW algorithm results visualization are far more usable than the popular DTW signal alignment plot (see Figure 5) because our plots inform us about real distances between aligned samples rather than indices of alignment samples. Indices are not very relevant if we want to compare two actions using the DTW framework. The evaluation of the proposed methodology is performed on a very large dataset that contains 28 various karate techniques performed by two elite karate masters. Each technique is repeated 10 times: the dataset contains in total 560 recordings. Half of this dataset can be downloaded from the website of our project [38]. The rest of it will be published in the same place after finishing the ongoing research grant.

Our methodology can be nicely applied to martial arts action analysis, because the practice of combat training requires sequential repetition of prearranged movements until their performance becomes nearly automatic. Athletes optimize their skills by repeating the template movements presented by the coach. The computer-aided analysis of this process is one of the possible applications of the methodology presented in this paper. What is more important, combat sports and MoCap analysis are influential on athletes’ health, injuries and rehabilitation [39,40]. Furthermore, data generated by our approach can be visualized in a virtual reality system that can be used as an innovative training procedure for athletes [41]. The proposed algorithms are composed of some established approaches; however, applying them according to our research idea resulted in valuable theoretical and practical results.

In the following sections, we will present the computational problem we are dealing with, the dataset and the proposed methodology.

## 2. Materials and Methods

In this section, we will describe our dataset, kinematic chain model and algorithms for template generation, matching, comparing and visualization applied to motion capture data.

### 2.1. Dataset and Kinematic Model

The MoCap data were recorded with a Shadow 2.0 wireless motion capture system. It consisted of 17 inertial measurement units that contain: 3-axis accelerometer, gyroscope and magnetometer. The information about range and resolution is presented in Table 1. The tracking frequency was set to 100 Hz with 0.5 degrees static accuracy and 2 degrees dynamic accuracy. This motion capture system has already been used and cited in scientific research; for example, [42,43]. Data measured by inertial sensors of our costume are processed by the software provided by the hardware manufacturer to calculate the above-mentioned MoCap signal values according to the hierarchical model in Figure 1. Each body joint of the model holds information about local rotations (orientation) of the so-called “bone” attached to it, relative to the parent joint. The root (hips) joint holds information about global rotation of the whole body. Those rotations are measured in Euler angles (this is the standard output format of the software). The calibration pose is a T-pose. In order to calculate the global reference frame, the MoCap system we used, similarly to other popular inertial solutions [44], acquires information regarding the location of the experiment and estimates the magnetic field of the Earth. These improve the determination of the global reference frame, enabling the correction of the angle between the gravity vector and magnetic field. After the calibration procedure, persons taking part in the recording might not face the same direction. As the body direction, we assume the rotation of the hip joint around the y (vertical) axis.

Numerous studies have reported successful use of systems based on accelerometers or gyroscopes in sports and medical applications [45,46]. Among the biggest advantages of this type of solution is the portability of the solution and the possibility to use it in indoor and outdoor environment. What is more, the obtained data do not require further manual post-processing, which is quite common in visual MoCap solutions, for example due to marker occlusion and confusion. Research also proved that it is possible to obtain comparable accuracy of inertial sensors with respect to the optical MoCap system [47,48]. The disadvantage is the lack of direct position tracking (this is calculated by double integration of accelerometer data) [49], which might cause some amount of drift. Despite this, in some circumstances, researchers prefer inertial technology over optical [50]. All of the mentioned factors might influence the final accuracy of tracking; however, the quality of our MoCap system seems to be suitable to evaluate our proposed methodology on karate data. Nevertheless, the proposed template generation algorithm works in the same way regardless of the motion capturing technique.

We have obtained data from two highly-skilled black belt karate athletes. One of them is a multiple Oyama karate knockdown champion, while the other is a Shorin-ryu kata champion. Both of them performed several karate techniques, and every technique was repeated 10 times. The Oyama karate master performed the following group of techniques:

- stances: kiba dachi, zenkutsu dachi, kokutsu dachi;
- blocks: gedan barai, jodan uke, soto uke, uchi uke;
- kicks: hiza geri, mae geri, mawashi geri, yoko geri;
- punches: furi uchi, shita uchi, tsuki.

All techniques were performed both on left and right side (leg or hand), giving in total 28 techniques.

The Shorin-ryu karate master performed the following group of techniques:

- stances: naihanchi dachi, shiko dachi, zenkutsu dachi, uki ashi dachi;
- blocks: age uke, soto uke, uchi uke, gedan barai;
- elbow strikes: age empi, mae empi;
- punches: choku tsuki;
- kicks: hiza geri, mae geri, mawashi geri, yoko geri.

The detailed movement descriptions with illustrations can be found, for example, in [51].

All techniques besides the fully-symmetrical ones like naihanchi dachi and shiko dachi were performed both on the left and right side (leg or hand), giving in total 28 techniques. All recordings are from 2–4 s in length. We have performed sensor calibration before each motion capture session using software provided by the hardware manufacturer. Because we acquired 10 samples of each karate technique of both athletes, we finally gathered 560 recordings. We have chosen those particular techniques to be recorded because they are commonly used during karate training. To precisely describe the movements, we used the kinematic chain model (see Figure 1), the root of which is the hips joint. The descriptions below show where sensors are placed on the subject.

- Head – located approximately between left and right temple, over eyebrows.
- Right/left shoulder: placed in the middle of the left scapula.
- Right/left arm: placed between the acromioclavicular joint and lateral epicondyle in a proportion 1/3–2/3 closer to the shoulder.
- Right/left forearm: placed between the lateral epicondyle and wrist in a proportion 1/3–2/3 closer to wrist.
- Right/left hand: approximately over the capitate carpal bone and third metacarpal bone.
- Chest: approximately on the xiphoid process of the sternum.
- Hips: between the left and right anterior superior iliac spine.
- Right/left thigh: over the lower lateral 1/3 surface of the thigh, just below the swing of the hand.
- Right/left leg: placed over the lower 1/3 of the shank.
- Right/left foot: placed on tarsal bones superior.

We utilize only angle-based notation of the chain to make the motion model invariant to body proportions. The algorithm does not use dual quaternions because we do not apply translations in our model. Ideally, all of the considered actions that are performed properly would be performed entirely identically, and the analyzed data would be equal among all observations [33]; however, there are always some deviations. The averaging was performed only on recordings that consisted of correctly performed actions. The decision whether the action was correct or not has been made by an expert.

### 2.2. Rotation Signal Averaging

MoCap data acquired by our system are a set of time-varying signals. We have recalculated Euler angle representation to quaternions. To average a set of time-varying signals, we have used the dynamic time warping barycenter averaging (DBA) heuristic algorithm [52] that exploits the DTW similarity measure. The cost function in DTW for quaternions is defined as [28]:

(1) $$cf\left(x,y\right)=1-\left|x\circ y\right|$$

where *x*, *y* are normalized quaternions, ∘ represents a dot product and ‖ is an absolute value. The absolute value is necessary because the unit quaternions q and −q correspond to a single 3D rotation.

There are several other functions that have been proposed to measure distance in a quaternion space, for example:

- the length of a geodesic line connecting two quaternions [26]:
  (2) $$dg\left(x,y\right)=2\cdot min\left(∥log(x^{-1}\cdot y)∥,∥log(y^{-1}\cdot x)∥\right)$$
  where ‖ ‖ is a quaternion norm.
- the distance in tangent space, into which quaternions are transformed by the logarithm operator [28]:
  (3) $$dtan\left(x,y\right)=∥log(x)-log(y)∥$$
- corresponding to rotation angle concatenation of rotations q1 and inverse rotation to q2 [28]:
  (4) $$drot\left(x,y\right)=re\left(x\cdot \bar{y}\right)$$
  where $\bar{y}$ is a conjugate quaternion.

Based on the experiment presented in [28], where the authors evaluated various distance functions in the task of gait identification based on the nearest neighbor classification technique with motion similarity assessment by dynamic time warping, we have chosen Function (1), which gave the best results while using all joints and was the least numerically complicated. Furthermore, visual evaluation of the initial results of application (2)–(4) to DTW done by an expert did not show superiority of any of them over (1).

Because we are dealing with quaternions, the barycenter averaging is replaced by Markley’s algorithm, which is a norm-preserving approach [53]. Markley’s algorithm is defined as:

(5) $$M=\left\{\begin{array}{c} M_{0}=\left[\begin{array}{cccc} 0 & 0 & 0 & 0\\ 0 & 0 & 0 & 0\\ 0 & 0 & 0 & 0\\ 0 & 0 & 0 & 0 \end{array}\right]\\ M_{i}=M_{i-1}+w_{i}\cdot q_{i}\cdot q_{i}^{T} \end{array}\right.$$

(6) $$\bar{q}=arg\max_{q\in S^{3}}q^{T}\cdot M\cdot q$$

where $S^{3}$ denotes the unit 3 sphere, $q_{i}$ the i-th quaternion to be averaged and $w_{i}$ the i-th weight from weight vector. The average quaternion $\bar{q}$ is the eigenvector of *M* corresponding to the maximum eigenvalue.

**Figure 2:**
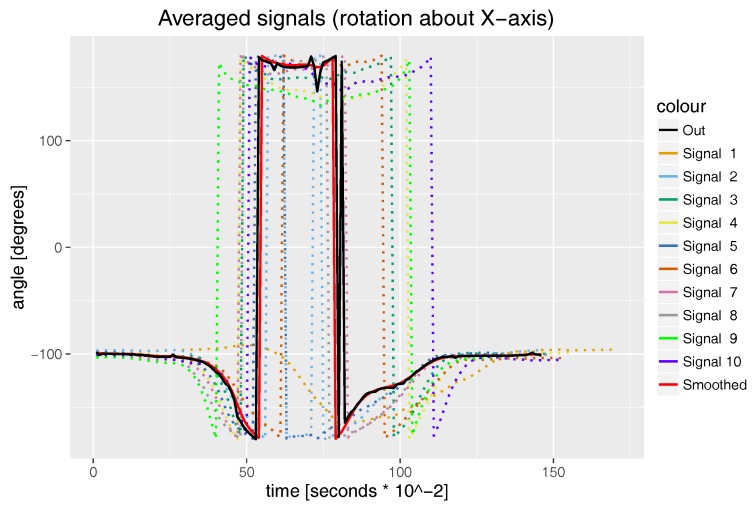
The results of the signal averaging and smoothing algorithm. The plot shows the rotation of hips joint about the *X*axis during mae geri kick with left leg. Dotted plots named Signal 1–Signal 10 are the input data. The solid black line (out) is a result of signal averaging, while the solid red line is the smoothed averaged signal.

We use the DTW approach because the activities we want to compare might be performed at different speeds and there might be nonlinear scaling differences between signals. For example, in the case of mae geri kick, the proportion of time between lifting a foot from the ground with simultaneous knee joint flexion in the first part of a kick and knee joint extension in the second part of the kick is not constant and might differ between recordings; see how this phenomenon affects the knee joint rotation in Figure 2. That means that simple resampling of all signals to have the same length (number of samples) and performing, for example, Markley’s algorithm directly on them will not result in valuable averaging results.

The proposed averaging algorithm is presented as pseudocode in Algorithm 1.

As we already mentioned, it is possible that persons in each pair of MoCap face different directions. As the body direction, we assume the rotation of the hip joint around the y (vertical) axis. In the hierarchical kinematic model, we can make all persons face the same direction by rotating only the root (hips) joint using the following approach.

Let us assume that we want a person to face direction:

(7) X←[1,0,0]

and we want to apply a rotation only by the y (vertical) axis. The problem we have to solve is to find quaternion φ that minimizes the following formula:

(8) $$\min_{\varphi }\left(R\left(\varphi ,R\left(\alpha ,X\right)\right)\circ X\right)$$

where R(β,Y) is a rotation transform of vector *Y* by quaternion β and α is an initial rotation of *X*. Equation (8) can be solved relatively fast by the brute-force search approach. 

**Algorithm 1:** Signal averaging algorithm. 
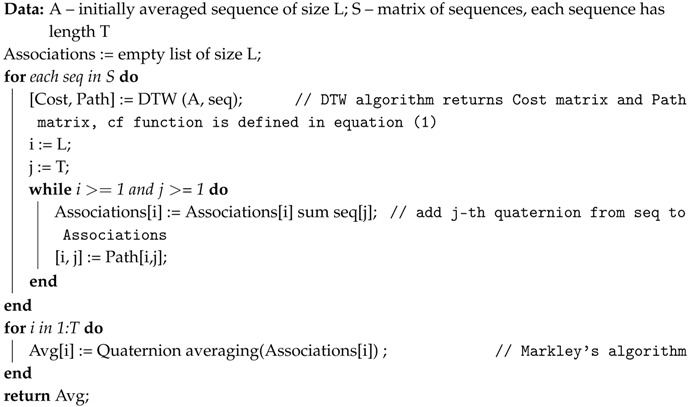


### 2.3. Signal Smoothing

The DBA algorithm might introduce in averaged signals high frequency noises that are visible as rapid Euler angle hops in Figure 2 (see the black “out” signal). This is of course the result of the DBA heuristic, which does not prevent these situations, even if input data do not contain that type of noise. Our proposed smoothing algorithm, which is an extension of [37], works similarly to the typical discrete linear convolution algorithm with the Gaussian kernel; however; instead of the linear combination of signal samples in the kernel window and kernel weights, we use once again Markley’s algorithm. The smoothing algorithm of discrete quaternion signal *Q* is defined as:

(9) $$Qs_{j}=arg\max_{q\in S^{3}}q^{T}\cdot M_{\left\{j-ws,\ldots ,j+ws\right\}}^{G}\cdot q$$

where $Qs_{j}$ is the j-th value of smoothed *Q*, *G* is a symmetric vector with a Gaussian distribution of values with length ws+1 and $M_{j-ws,\ldots ,j+ws}^{G}$ is Matrix (2) constructed from ws+1 quaternions of *Q* width indexes {j−ws,…,j+ws} and weight vector G. The smoothing results are presented in Figure 2 (see the red “smoothed” signal).

The proposed averaging algorithm is presented as pseudocode in Algorithm 2.

**Algorithm 2:** Smoothing algorithm. 
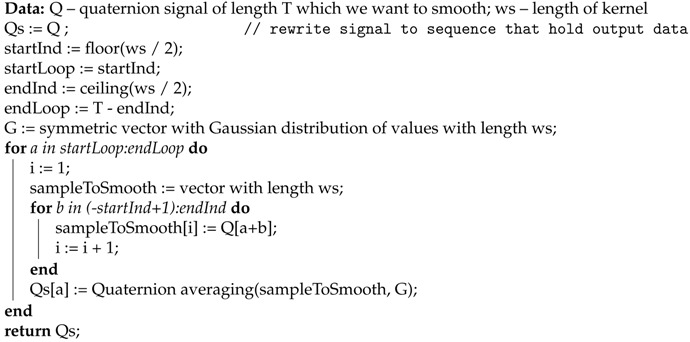


### 2.4. Full-Body Template Generation

To generate the full-body template of an action, we use all MoCap recordings of the given type as input data. The Euler angles are recalculated to quaternions (both global rotation hips joint angle and all local angles). This step removes problems caused by discontinuity in rotation descriptions in the Euler angle domain [−180,180) for rotation around the *X* axis, [−90,90) for *Y* and [−180,180) for *Z*. In the situation when we are using Euler angles to describe joint motion in the domain we mentioned earlier, there might be an event while performing an action when a “bone” rotation around an axis causes the discontinuity of the rotation plot (see the dotted lines in Figure 2 where if the angle is less than −180 degrees, it is flipped to be close to, but not to overcome 180 degrees). These discontinuities might not appear in all signals that represent the same action; for example, Signal 1 does not have it; however, the joint rotation driven by it does not differ much from other signals. Those discontinuities that are present in the Euler angle domain might cause averaging algorithm failure. To overcome this, we decided to use quaternions, where this phenomenon is absent. What is more, quaternions prevent the gimbal lock phenomena and guarantee that the averaged signal retains all degrees of freedom. We apply (8) to correct the rotation of hips joints and then Algorithms 1 and 2 to each quaternion signal separately. The averaged and smoothed quaternions are recalculated to Euler angles once again and integrated into single MoCap. This last quaternion to Euler angle conversion is done to facilitate visual evaluation of the results. The computational complexity of our algorithm (ALG) equals:

$$O\left(ALG\right)=O\left(SN\cdot Iter\cdot O\left(DTW\right)\right)=O\left(SN\cdot Iter\cdot Len^{2}\right)$$

where: SN is the number of sequences; Iter is the number of iterations; Len is the signal length. As can be see, the computational complexity is polynomial, and the algorithm can operate in real time. By “real time” in this context, we mean that: as the algorithm has a polynomial complexity, it can be applied to a given time window, and the averaged signal might be computed in time comparable to the acquisition frequency of typical professional MoCap hardware. DTW commonly uses a sample of a certain length to calculate the warping path. Because of that, in the typical scenario, DBA, which uses DTW, will not run on successive samples, but rather on selected parts of previously acquired data.

### 2.5. Signal Matching and Visual Analysis

**Figure 3:**
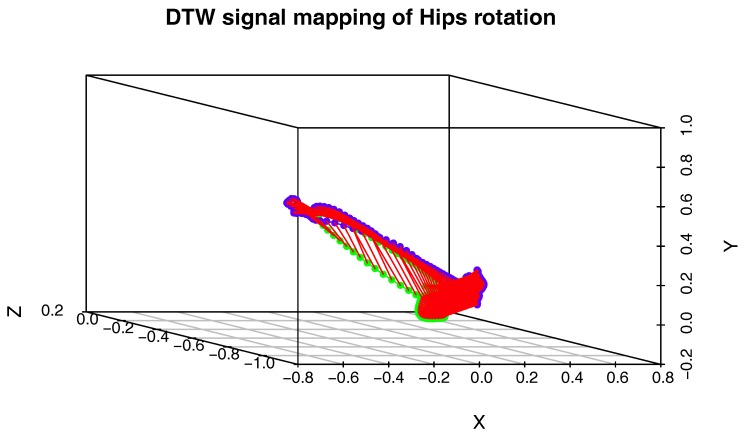
DTW signal mapping of hips rotation between template (green signal) and one selected MoCap recording (blue signal) of mae geri kick with the left leg. Those are the same MoCap as in Figure 5. Distances are presented as red sections.

To compare two actions on the level of a single joint, we will use a procedure that generates the three-dimensional plot of the DTW signal mapping using Measure (1). We use this approach because the activities we want to compare might be performed with different speeds, and there might be nonlinear scaling differences between signals. To solve the problem with various body proportions, we represent the movement only with quaternion angles. The generation of the DTW signal mapping plot is presented in pseudocode in Algorithm 3. This time however, we do not need cost and path matrices, but matching vectors of Signal 1 onto Signal 2 and vice versa, which are also generated by DTW. An example plot of this type is shown in Figure 3.

**Algorithm 3:** DTW signal mapping plot generation algorithm. 
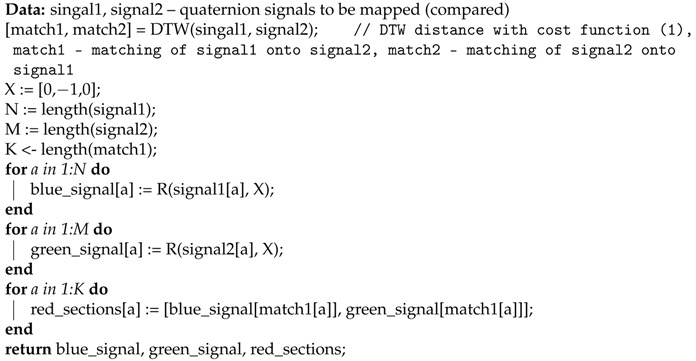


To present distances between two DTW matched signals, one can also plot the following two-dimensional drawing composed of the following components:

- The first signal is represented as a straight, vertical line with *n* sampled points marked on it (*n* is the signal length).
- The second signal is plotted over the first one. It consisted of *m* sampled points (*m* is the signal length). The vertical coordinates of each point are proportional to the DTW distance between this point and the corresponding point of the first signal.
- It has sections that show DTW mapping (corresponding points according to DTW) between both signals.

**Figure 4:**
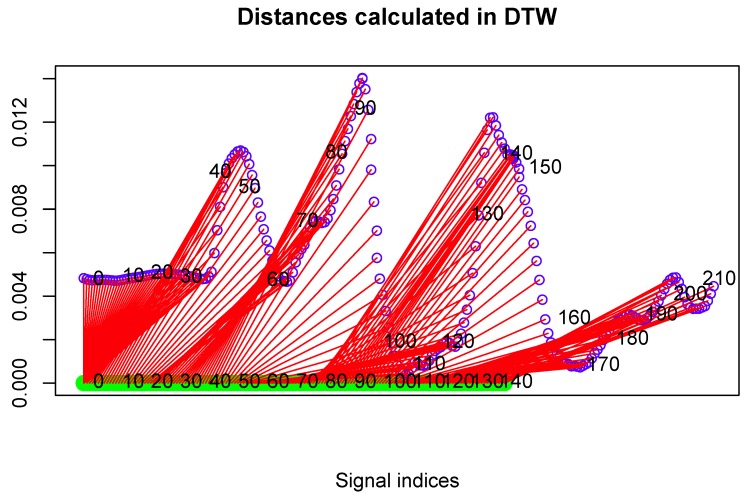
Distance of hips rotation between template (green signal) and one selected MoCap recording (blue signal) of mae geri kick with the left leg. Those are the same MoCap as in Figure 5. Distances are presented as red sections.

An example plot of this type is shown in Figure 4.

## 3. Results

The basis of our averaging method is the DBA algorithm, an averaging method which iteratively refills an initially (potentially arbitrary) selected sequence, in order to minimize its squared distance (DTW) to averaged sequences. As it is a heuristic, it does not guarantee that the obtained solution will be optimal. Our experiments are designed to evaluate if our approach can be utilized for some specialized class of time-varying signals, namely multidimensional MoCap recordings. As our dataset is large (560 recording) and contains a wide range of various techniques (28 classes), we believe that this sample is representative to prove the validity of our methodology. We have prepared and performed several experiments designed to examine the following key aspects of our methodology:

1. Evaluating the quality of generated templates: By quality, we mean the averaging potential of the proposed method and the correspondence of template 3D rendering to the original MoCap recordings. That means that the template has to be not only similar to the input data in the numerical sense, but it also has to present the correct karate technique.
2. Evaluating the potential of the matching algorithm to calculate similarities and differences between various MoCap data.
3. Applying generated templates to human action classification.
4. Evaluating visualizations of important difference and similarities between MoCap data detected by the previous methodology.

The first experiment was performed on the whole dataset introduced in Section 2.1. We have generated templates of all techniques performed by Oyama and Shorin-ryu karate masters separately. Due to the paper space limitation, we cannot present complete evaluation results, so we decided to show them aggregated in the form of various plots.

Renderings of MoCap templates we obtained show the correct performance of all techniques. In the Supplementary Materials, we present two example renderings of MoCap templates. The renderings are visualizations of bvh (Biovision Hierarchy) files we generated with our algorithm. Visualizations were done in Blender 2.69 and played four-times slower than the original data. Ten figures in the rear row are the input (original) Mocap recordings, while the figure in front of them is the result of averaging. The first visualization contains mae geri kick with the left leg of the Shorin-ruy karate master, while the second mawashi geri kick with the left leg of the Oyama karate master.

In Figure 2, we present the example averaging and smoothing calculated by our method. Figure 6 and Figure 7 summarize our experiment and show bar plots of median values of dynamic time warping normalized distances calculated for each karate technique. The smaller the values of normalized distances, the more similar is a generated template to the recordings from which it was calculated. Due to the large variance of results caused by joints that are not relevant for a given action (for example, hands during kicks), we use the median value, which discriminates outlier values, instead of the average value. The differences in median DTW normalized distances presented in Figure 6 and Figure 7 show the general intuitive rule that a higher median occurs in moves that are more complex and requires larger mobility of the body. For example, it is easier to performer a similar stance than to perform a similar kick or punch.

The more detailed results of this evaluation are presented in Figure 8. It shows in the form of a matrix dynamic time warping normalized distances between template and input signals of mae geri front kick with the left leg of the Shorin-ryu karate master. This matrix demonstrates the results of averaging. The bigger the difference is between the particular signal and template, the higher DTWND (dynamic time warping normalized distances) we obtain. The relatively high DTWND values might be present on the map because of two cases. In Figure 8, we see only relatively high differences in left thigh joints in comparison to other joints. This means that movements of the rest of the body are relatively similar between cases, and differences are present because, as we already mentioned, in practice, it is impossible to perform some action ideally; there are always deviations. The highest deviations happened in the joints that required the highest mobility. The second reason for the differences occurs when the matrix visualizes data from two different actions. If one action is a correct template and the other action we want to examine is not, the matrix indicates in which part of the body actions differ the most. The body joints discovered that way should be the target of further analysis, for example by Algorithm 3; we will discuss this case later.

The aim of the second experiment was to compare a template generated with our approach from actions of one person with actions that were performed by another person. We took the template of the Shorin-ryu karate master that performed mae geri kick with the left leg standing in zenkutsu dachi stance. We compare it to mae geri performed with the left leg from kumite-no-kamae stance of the Oyama master (the initial stances differ from each other). We decided to choose this particular technique because it characterizes not only high dynamics, like all karate attacks, but also it has a large radius of leg movement. What is more, the height of the kick might differ between even the most skilled athletes because it is conditioned by the flexibility of particular persons and their anatomy. Due to this, the proposed evaluation will examine not only the possibility of aligning very similar MoCap data, but also the possibility of dealing with various differences that are present on recordings of people performing the same body action.

**Figure 5:**
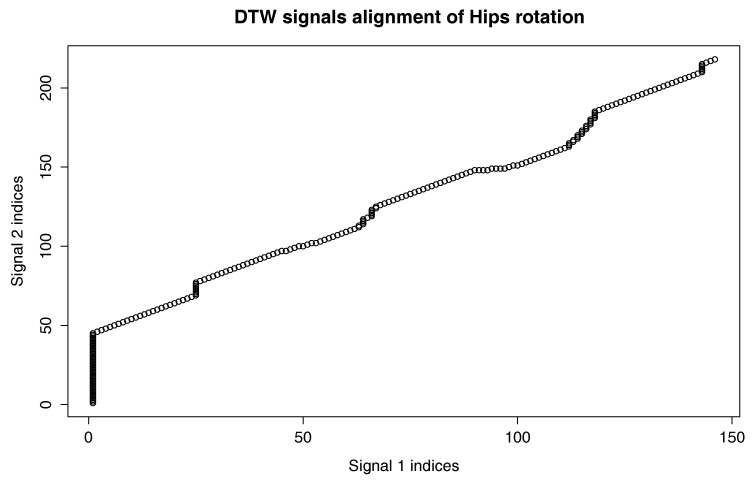
DTW signal alignment of hips rotation between the template (Signal 1) and one selected MoCap recording of mae geri kick with the left leg (Signal 2). The template was generated from the Shorin-ryu karate master data, while the other MoCap came from Oyama karate master.

Figure 9 presents the comparison of those two MoCap data we mentioned earlier in the form of a matrix. We skipped signals that are irrelevant for comparison, like head and hand positions. In the next Figure 3, Figure 4 and Figure 5, we evaluate the DTW aligning and mapping (with Algorithm 3), and we plot the DTW distance of the hips joint.

We have used data visualized in Figure 4 to find MoCap frames that represent the most distant (local maxima) and least distant (local minima) corresponding points. They allow us to identify the corresponding parts of actions that are least and most similar to each other.

The motion template generated by our method can be used for action recognition purposes. The goal of human action recognition is to assign the recording presenting a person performing some activity to one of the predefined classes. There are many methods to register human movements for example by depth cameras and inertial sensors [34,54,55], and the registration method partially determines the possible classification algorithm. Because we generate a single action template that is averaged from several recordings, the straightforward approach that can be used to classify an action is the DTW classifier [55]. This type of classifier contains templates of actions and compares them with recording to be recognized. This recording will be assigned to the class, to which the corresponding template has the smallest DTW normalized distance value. In order to make the classification effective, we have to select the appropriate feature set. Based on our experience with karate action recognition [56,57], we will use angle-based features between the axis of the coordinate system that is relative to the body position and vectors designed by coordinates of body joints. As we already mentioned in [57], when we are dealing with whole body action recognition of very similar actions that differ only by some nuance (like various types of kicks or punches), it is better to use different feature sets for various action groups. That is because when we use too many feature, there will be too many constraints for actions to be correctly classified. On the other hand, where the number of features are too small, there will not be a possibility to distinguish between similar actions (like for example various karate kicks). Because of those factors, it is very difficult to create a single feature set that can be successfully used to classify various movement tasks. In the rest of this paper, we will consider a feature set that can be used to classify karate kicks (lower part of the body).

The unnormalized vectors of the coordinate system are defined as:

(10) $$\left\{\begin{array}{c} \bar{X}=LeftThigh-RightThigh\\ \bar{Y}=Chest-Hips\\ \bar{Z}=XxY \end{array}\right.$$

where the names of the vectors correspond to the names in Figure 1 and *x* is a cross-product. The three-dimensional coordinates of the features have been recalculated from the hierarchical to the kinematic model. We also use four vectors defined as:

(11) $$\left\{\begin{array}{c} \bar{V1}=LeftLeg-LeftThigh\\ \bar{V2}=LeftLeg-LeftFoot\\ \bar{V3}=RightLeg-RightThigh\\ \bar{V4}=RightLeg-RightFoot \end{array}\right.$$

We have used twelve angle-based features that are angles on the plane:

(12) $$\left\{\begin{array}{c} F1=∡\left(\bar{X},\bar{V1}\right);F2=∡\left(\bar{Y},\bar{V1}\right);F3=∡\left(\bar{Z},\bar{V1}\right)\\ F4=∡\left(\bar{X},\bar{V2}\right);F5=∡\left(\bar{Y},\bar{V2}\right);F6=∡\left(\bar{Z},\bar{V2}\right)\\ F7=∡\left(\bar{X},\bar{V3}\right);F8=∡\left(\bar{Y},\bar{V3}\right);F9=∡\left(\bar{Z},\bar{V3}\right)\\ F10=∡\left(\bar{X},\bar{V4}\right);F11=∡\left(\bar{Y},\bar{V4}\right);F12=∡\left(\bar{Z},\bar{V4}\right) \end{array}\right.$$

The distance function for DTW is defined as the Euclidean distance between the vector of features [F1,F2,F3,F4,F5,F6,F7,F8,F9,F10,F11,F12]. Using this features set, we have performed leave-one-out action recognition for the Shorin-ryu and Oyama karate master separately. We have taken into account mawashi geri, mae geri, hiza geri and yoko geri (both with the right and left leg), so we have eight classes of actions. Templates were generated from nine motion recordings, and the remaining one was classified using the template. In this experiment, each action was correctly assigned to its class. In another experiment, we used templates generated from Oyama master recordings to classify the Shorin-ryu master recordings and vice-versa. Because the Oyama master performed yoko geri kicking sideways, while the Shorin-ryu master kicked in front, those two motions could not be compared because they create different templates. For both athletes, all mawashi geri (with right and left leg) and all but one mae geri kick were correctly classified. One mae geri kick with the left leg was assigned to the hiza geri left class. Three hiza geri kicks with the right and three with the left leg of the Shorin-ryu master were classified as mae geri kicks. The overall recognition rate was 94.2%, which is a very good result for this type of complex action. The mae geri and hiza geri kicks can be misclassified because hiza geri (knee strike) and mae geri (straight kick) have nearly the same thigh trajectory, and also shin movement at the beginning and end of those actions is very similar. The detailed results are presented in Table 2.

The angle-based feature setup we use in this experiment is often chosen by other researches (see for example the state of the art presented in [13]). Although using quaternions is among the most convenient ways for processing three-dimensional rotations in advanced signal processing (we explained this in Section 2.4), in the case of actions, recognition features based on Euler angles might be a better choice due to several reasons. At first, in many cases, to successfully perform action recognition, we do not need to use features that represent complete motion of the whole body (like 3D rotations in quaternion form); rather some motion derivatives that are sufficient for the action class we want to classify. For example, for classification purposes, we can replace four-dimensional quaternions by three-dimensional Euler angles on the planes between vectors of the coordinate frame and vectors representing limbs. If we decide to use this simplified approach, the feature set for each body joint has one dimension less compared to quaternion notation (which is four-dimensional). In our case, for example, instead of representing each sample with a 16-dimensional vector using quaternions, we have 12-dimensional vectors of Euler angles. This feature definition makes classification less constrained. The second reason is that results obtained with angles on the plane are easier to interpret than quaternions, because when we calculate angles between vectors using the dot product, they are in domain [0,180]. Furthermore, various classes of actions described by angle-based features can be nicely visualize in three-dimensional space using PCA (see for example [57]).

The last experiment evaluates how the proposed matching method behaves when we use it to compare two different techniques that have however some similarities. To complete the tasks, we have chosen the same mae geri (front kick) template as in the previous experiment; however, we compared it with mawashi geri (roundhouse kick) of the Oyama karate master. It is obvious that the front kick has a different hips trajectory than the roundhouse kick; however, the knee joint (relative to the parent joint) rotates in a similar manner. The evaluation was performed in the same manner as in the previous experiment, and results are visualized in Figure 10, Figure 11, Figure 12 and Figure 13.

The matrix in Figure 10 visualizes the essential differences between two correctly-performed karate techniques: the front kick and the roundhouse kick. While the position of the upper parts of the body remain relatively similar (spine low, spine mid and chest joint), the highest differences are in the movements of the left and right thighs (thigh rotation propels the impact of the kick). What is more, in the case of the roundhouse kick, the athlete also twists the leg that is not used for kicking (in the case the left leg kick, it is a right leg) to increase the range of thigh rotation. The presence of those rules is clearly visible on the obtained matrix. The lack of them would mean that one of those kicks is not performed correctly. For example, insufficient body rotation in the roundhouse kick is a common problem of the young karate students. Without it, the roundhouse kick becomes too similar to the front kick and thus ineffective.

## 4. Discussion

The results presented in the previous section prove that all proposed methods work as expected and are easy to use and robust algorithms. The proposed signal averaging method is a combination of the DBA algorithm with Markley’s algorithm performed on quaternions. As can be seen in Figure 2, the smoothing algorithm deals very well with high frequency noises. This can be clearly noticed when comparing a black (out) signal with the red (smoothed) one, which has a nearly identical shape, however without high frequency “leaps”, which are the result of the DBA heuristic. Our smoothing algorithm is basically very similar to the typical low-pass convolution filter that uses the Gaussian kernel; however, instead of the weighted summation of values in the filter window, we used Markley’s approach. Human motion recordings done with a high-frequency MoCap system (like ours) do not contain signal samples with values highly deviating from adjoining samples like those visible in Figure 2 after $75\times 10^{-2}$ s (it is virtually impossible for a human being to move that fast). Due to this, we can safely assume that this is a signal disruption introduced either by the MoCap hardware or DBA algorithm, and it should be filtered out. The application of the quaternion representation of angles allows us to overcome the problems of discontinuity in rotation descriptions in Euler angles.

**Figure 6:**
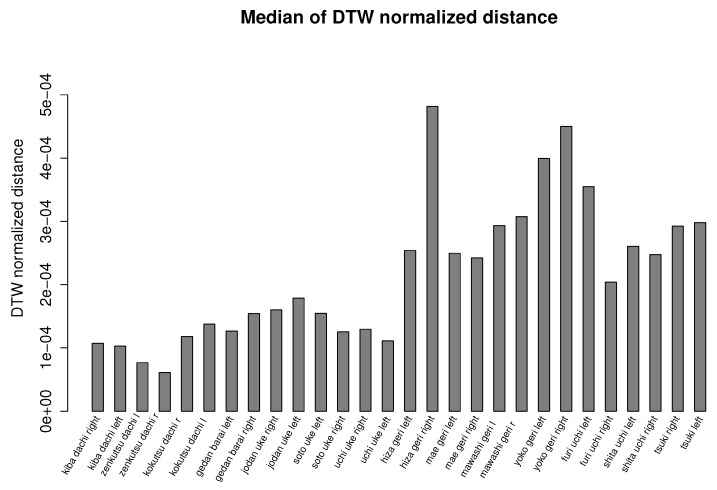
Bar plot of the median value of dynamic time warping normalized distances calculated for each karate technique of the Oyama karate master. At first, the dynamic time warping normalized distance between template and input signals is calculated, then we take the median value of all calculated distances.

**Figure 7:**
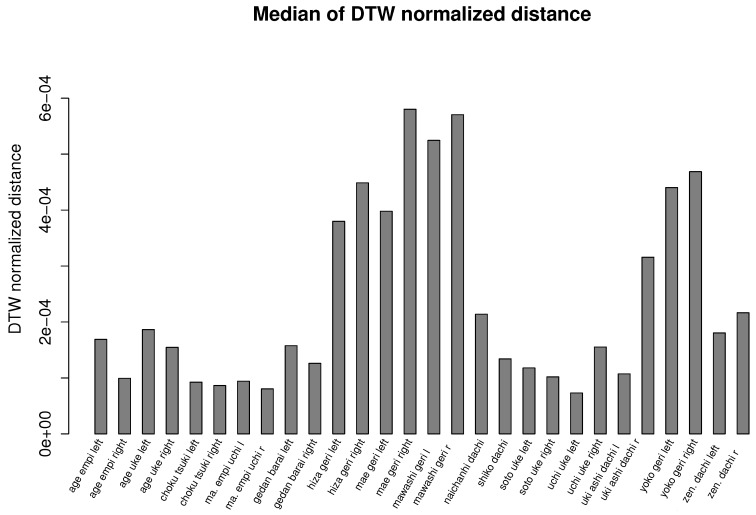
Bar plot of the median value of dynamic time warping normalized distances calculated for each karate technique of the Shorin-ryu karate master. At first, the dynamic time warping normalized distance between template and input signals is calculated, then we take the median value of all calculated distances.

**Figure 8:**
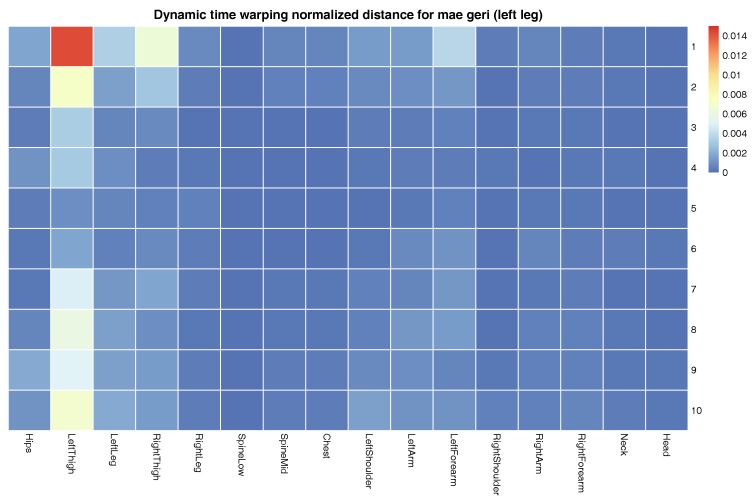
Dynamic time warping normalized distances between the template and input signals of mae geri with the left leg of the Shorin-ryu karate master. Each signal is presented in different rows, while each column is for different joint. Distances are presented on the matrix with colors. Each row represents a single take of the same technique.

The overall quality of templates measured as the median value of dynamic time warping normalized distances calculated for each karate technique also returns very good (low) results. The median distance for each technique does not overcome the order of $10^{-4}$ (see Figure 6 and Figure 7). In the case of recordings about 2–4 s in length that we are dealing with, this distance means that the differences between MoCap data nearly cannot be spotted by visual observation. Of course, the lengths of MoCap between template and input data might differ; however, we are nearly sure that we generated the same action. This fact was confirmed by our observation. The more detailed results are presented in Figure 8. As can be observed, most of the DTW distances between template and input data have very small values. Most often, the larger distances are in the joint that moves the most; in the case of mae geri, it might be for example the thigh. However, in the case of highly-skilled class athletes, those differences are very low because they have high repeatability of movements.

**Figure 9:**
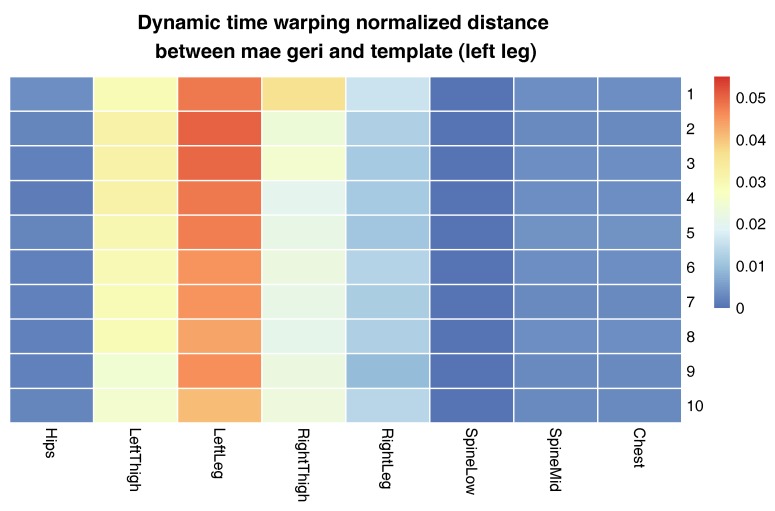
Dynamic time warping normalized distances between the template and input signals of mae geri with the left leg. The template was generated from the Shorin-ryu karate master data, while other MoCap came from the Oyama karate master. Distances are presented on the matrix with colors. Each row represents a single take of the same technique.

**Figure 10:**
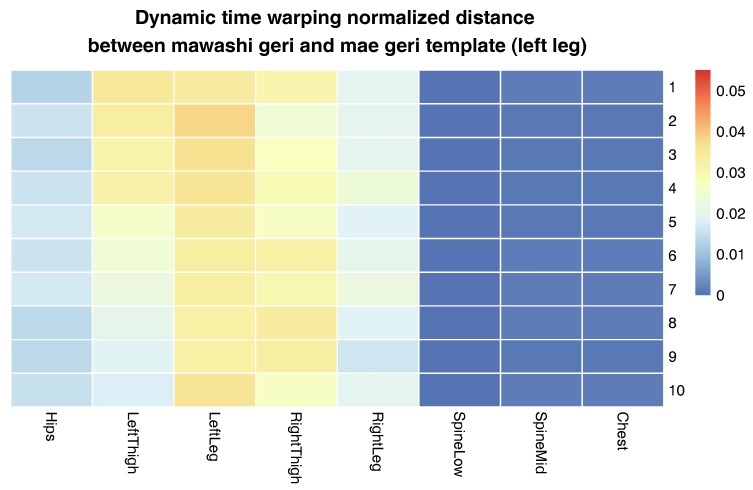
Dynamic time warping normalized distances between the template of mae geri kick with the left leg and the mawashi geri MoCap. The template was generated from the Shorin-ryu karate master data, while other MoCap came from the Oyama karate master. Distances are presented on the matrix with colors.

In the second experiment, we have compared the template of the same technique (mae geri) generated for two different karate masters. We wanted to find out if the proposed aligning metrology allows us to detect the most similar and least similar parts of the MoCap. We want to do it in order to generate the visual feedback for human action data comparison at a very precise level. The first important step in this type of comparison is to calculate the normalized DTW distance between the template and MoCap we want to compare and to visualize it for example in the form of a matrix (see Figure 9). The output of our algorithm that is presented as a matrix (Figure 9) or more detailed DTW signal mappings (Figure 3, Figure 4 and Figure 5) speed up the analysis of motion capture renderings. Matrices indicate which body joints should be compared, while DTW mappings allow us to detect frames that correspond to local maximums (top row) and local minimums (bottom row) of distances (Figure 10). All of these methods might be a part of the computer-aided training system giving additional feedback to the coach. Of course, like in all computer-aided systems, the final decision if some differences are relevant from the perspective of the martial art and should be eliminated to improve the techniques is made by the trainer.

We can spot that the biggest differences are between leg (thigh) joints. Differences are caused by different flexibility (or height of the kick) of athletes and different initial stances. However, the most interesting for us is the hips joint that drives the direction of the whole kick. Figure 9 shows that there are not many differences between the template and MoCap of other athlete. In order to make a deeper analysis, we can generate a DTW signal alignment plot (see Figure 5). In this kind of plot, if its direction is more or less diagonal, we know that one signal is warped to another one smoothly. In our case, the first 50 frames of Signal 2 are wrapped to the first frame of Signal 1. That is because the initial stance of the second MoCap differs, and after 0.5 s, the hips position becomes more similar. The rest of the DTW proceeds smoothly. DTW signal mapping in Figure 3 allows us to visualize in 3D how one signal is aligned with another. It may be difficult to follow the signal trajectories when the image is printed on paper; however, this plot is very usable when one can rotate it around all axes and manipulate the number of points on the green and blue plot that are visualized. Thanks to this, we can clearly see the progress of movement and spot the differences and similarities in distance between signals and the timing of an action. When we are limited to two-dimensional visualization, the distance DTW plot presented in Figure 4 is far more understandable. Nevertheless, we have more complex information in Figure 3 because in Figure 4, both signals are “straightened”, and the biggest emphasis is put on distances between points in the template signal to corresponding points in the second plot. Analyzing local maxima and minima, we can find out which parts of the actions are most and least similar. In Figure 14, the visualization in the first row, which contains the rendering of frames within the local maxima (biggest differences), assures us that the differences are caused by different hips trajectories. That is the result of different initial and final legs positions of both karate masters. However, the most important parts of the mae geri kick action visualized in the bottom row seems to be nearly identical, although the Shorin-ryu master does not lift the leg as high as the Oyama master. In other words, those kicks were performed at different heights. That all proves that the proposed procedure can be easily applied for matching, comparing and visualization of similar actions.

**Figure 11:**
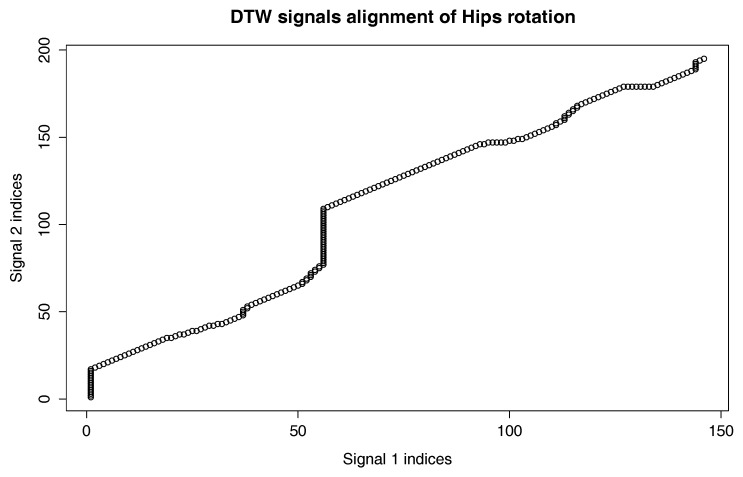
DTW signal alignment of hips rotation between the template of mae geri kick with the left leg (Signal 1) and one selected MoCap recording (Signal 2) of mawashi geri kick. The template was generated from the Shorin-ryu karate master data, while the other MoCap came from the Oyama karate master.

**Figure 12:**
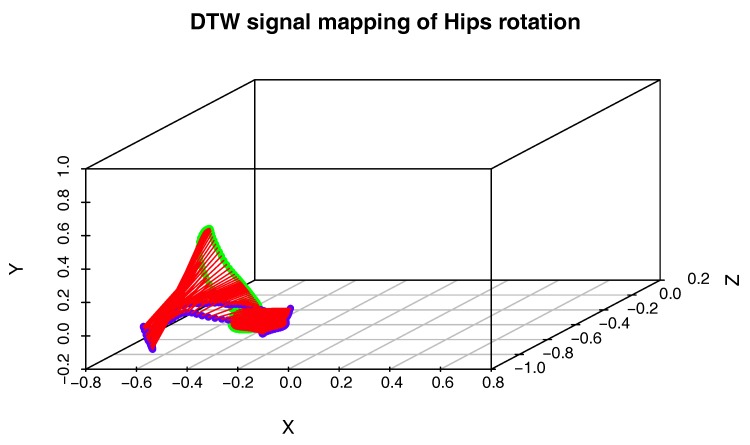
DTW signal mapping of the hips rotation between the template of mae geri kick with the left leg (green signal) and one selected MoCap recording (blue signal) of mawashi geri kick. Those are the same MoCap as in Figure 11. Distances are presented as red sections.

**Figure 13:**
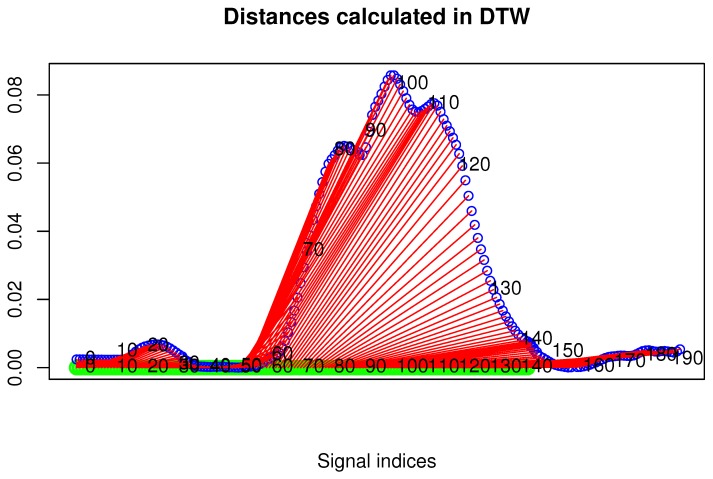
Distance of hips rotation between the template of mae geri kick with the left leg (green signal) and one selected MoCap recording (blue signal) of mawashi geri kick. Those are the same MoCap as in Figure 11. Distances are presented as red sections.

**Figure 14:**
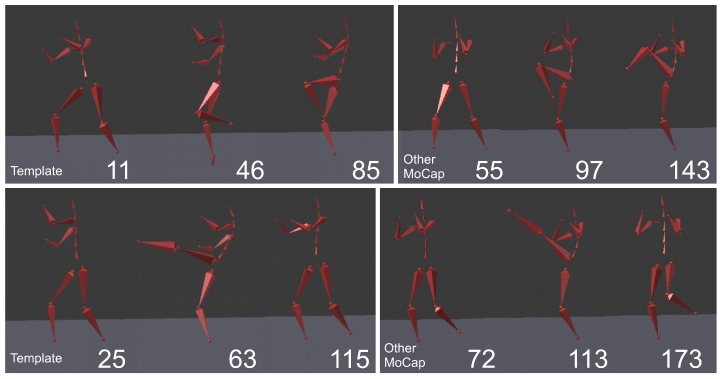
MoCap frames that correspond to local maximums (top row) and local minimums (bottom row) of the distances presented in Figure 4. Numbers that are present close to each visualization are indices from Figure 4.

The third experiment is similar to the previous one; however, this time, we have compared the template of the front kick with the MoCap of the roundhouse kick. The matrix indicates this time higher differences than previously in the hips DTW aligning. The differences are very clearly visible in the DTW signal alignment plot (Figure 11), where there is a large section that was not proportionally aligned in the middle of the recording (about 60 frames of Signal 1). That is because the axis of rotation goes in a different plane, which is clearly visible in Figure 12. In the case of mae geri, it goes in the vertical plane (green signal), while in mawashi geri, it goes in the horizontal plane (blue signal). The trajectory difference is also visualized in Figure 13. We can easily see without any MoCap rendering that we are dealing with actions that have totally different hips trajectories that are most similar only in the initial body position. That evaluation proves that the proposed algorithm works very well in comparing actions that are not similar to each other.

The results of our experiment might be influenced by the errors generated by the inertial MoCap system we used. Those might be both static and dynamic measuring errors. Various researchers report that while internal systems have good absolute static accuracy, clinically acceptable absolute accuracy under the condition of slow motion, the absolute and relative accuracies are significantly affected by velocity during sustained motions [44,58]. Those potential errors might be a source of additional differences between acquired body actions that were later used to generate the single template. If the introduced errors are random, the DBA algorithm might decrease their impact on the resulting template. The systematic error cannot be overcome by DBA. Furthermore, the environmental conditions might perturb the measurements to some extent, although we did not detect any electromagnetic disturbances with the electromagnetic field meter in the gymnasium where data acquisition had been performed.

## 5. Conclusions

Based on the results discussed in the previous section, we can conclude that our proposed methodology can be easily applied to human actions analysis, that is to template generation, matching, comparing and visualization. It works the best when we are dealing with relatively short actions (from 2–4 s) that might be averaged and aligned with the DTW framework. In practice, the methodology is designed to optimize the performance of some full-body techniques performed in various sports, for example combat sports and martial arts [59]. We can also use it to generate templates or to compare the correct performance of techniques between various top sportsmen to generate a knowledge base of reference MoCap videos. This type of knowledge base might be a very important source of information for coaches and athletes. The averaged sequences generated by our algorithm might be directly applied in the classification algorithms that use DTW.

In further research, we want to use our methodology to deeply examine the movement repeatability of athletes at different levels of advancement. With a large enough database, we will be capable of drawing conclusions about numerical values returned by our method; for example, what is an accepted variance of DTW distance between the template and MoCap data to consider actions similar and performed correctly. We also want to apply the comparison and visualization potential of our algorithm to aid in the process of training.

## Figures and Tables

**Table 1:** This table presents information about the inertial sensors of our motion capture (MoCap) system.

Sensor	Full-Scale Range	Resolution
Accelerometer	±2,4,8,16 g	0.18 mg
Gyroscope	±4000 °/s	0.12 °/s
Magnetometer	±8 gauss	0.25 mG

**Table 2:** Classification results of the DTW classifier on various karate kicks. Each row represents the correct action class; columns represent classification results. Because there are 10 recordings of each class for the Oyama and Shorin ryu masters, each class has together 20 motions recordings.

	Mawashi Geri Right	Mawashi Geri Left	Mae Geri Right	Mae Geri Left	Hiza Geri Right	Hiza Geri Left
**Mawashi Geri Right**	20					
**Mawashi Geri Left**		20				
**Mae Geri Right**			20			
**Mae Geri Left**				19		1
**Hiza Geri Right**			3		17	
**Hiza Geri Left**				3		17

## Supplementary Materials

The following are available online at www.mdpi.com/1424-8220/17/11/2590/s1, 1.mpg: mae geri kick with the left leg of the Shorin-ruy karate master; 2.mpg: mawashi geri kick with the left leg of the Oyama karate master.
